# Supplementary material for: Structural delineation of potent transmission-blocking epitope I on malaria antigen Pfs48/45
Source: Nat Commun. 2018 Oct 26;9:4458. doi: 10.1038/s41467-018-06742-9 (PMC6203815; doi:10.1038/s41467-018-06742-9)
Supplement: Supplementary file 1 — Supplementary Information [file 41467_2018_6742_MOESM1_ESM.pdf]

Supplementary Information

Structural delineation of potent transmission-blocking epitope I on malaria antigen Pfs48/45

Kundu, Semesi *et al.*

Supplementary Figure 1

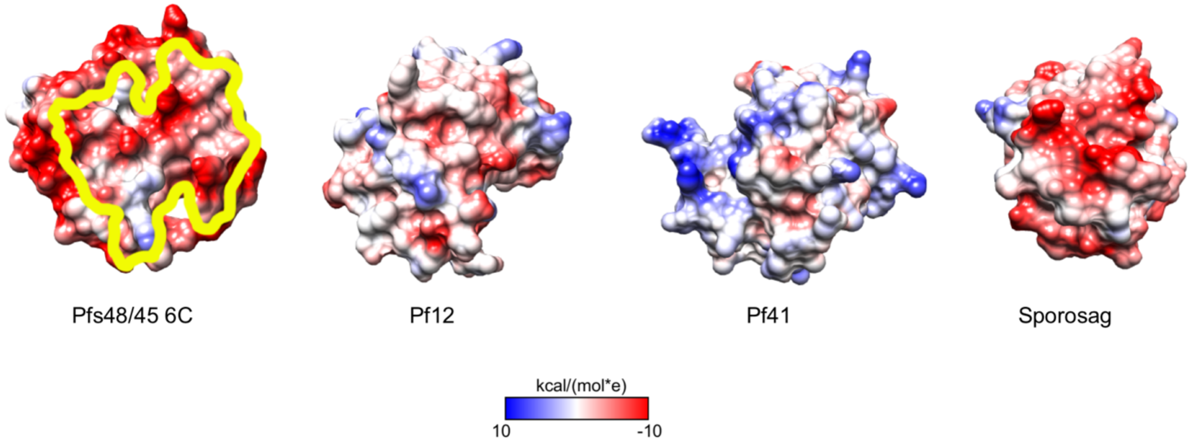

Surface properties of Pfs48/45 and homologous proteins. Coulombic electrostatic surface coloring of Pfs48/45 6C and domains of highest structural homology (Pf12, Pf41 and Sporosag; PDB ID's: 2YMO[1], 4YS4[2], 2WNK[3], respectively). The 85RF45.1 epitope (yellow outline) is predominantly electronegative and unique to Pfs48/45.

Supplementary Figure 2

A

```
Light chain candidate sequences

85RF45.1      1 QFVLSQPNSVSTNLGSTVKLSCKRSTGNIGSNYVSWYQHHEGRSPTTMIYRDDQRPDGVP
LC1           1 NFMLTQPHSVSESPGKTVTISCTRSTGNIGSNYVSWYQQRPGSSPTTVIYRDDQRPSGVP
LC2           1 QFVLTQPHSVSESPGKTVTISCKRSTGNIGSNYVSWYQQRPGSSPTTVIYRDDQRPSGVP
LC3           1 QFMLTQPHSVSESPGKTVTISCTRSTGNIGSNYVSWYQQRPGSAPTTVIYRDDQRPSGVP
IGLV6-57      1 NFMLTQPHSVSESPGKTVTISCTRSSGSIASNYVQWYQQRPGRAPTTVIYEDNQRPSGVP

85RF45.1     61 DRFSGSIDRSSNSALLTIDNVQTEDEAAYFCHSYSTGMYIFGGGTKLTVL
LC1          61 DRFSGSIDRSSNSASLTISGLKTEDEADYYCHSYSTGMYIFGGGTKLTVL
LC2          61 DRFSGSIDRSSNSASLTISGLKTEDEADYYCHSYSTGMYIFGGGTKLTVL
LC3          61 DRFSGSIDRSSNSASLTISRLKTEDEADYYCHSYSTGMYIFGGGTKLTVL
IGLV6-57     61 DRFSGSIDSSSNSASLTISGLKTEDEADYYC

Heavy chain candidate sequences

85RF45.1      1 EVQLVESGGGLLQPGRSLKLSCVASGFTFNNYWMSWIRQAPGKGLEWIASISNIGGTIYY
HC1           1 EVQLVESGGGLVQPGGSLRLSCAASGFTFNNYWMSWVRQAPGKGLEWVASISNIGGTIYY
HC2           1 EVQLVESGGGLVQPGGSLRLSCAASGFTFNNYWMSWVRQAPGKGLEWVASISNIGGTIYY
HC3           1 EVQLVQSGGGLVQPGGSLRLSCAASGFTFNNYWMSWVRQAPGKGLEWVSSISNIGGTIYY
IgVH3-7       1 EVQLVESGGGLVQPGGSLRLSCAASGFTFSSYWMSWVRQAPGKGLEWVANIKQDGSEKYY

85RF45.1     61 PDSVKGRFTISRDSAQNTLYLQMNSLRSEDTATYYCTRDLRMSDYFDYWGQGVMVTVSS
HC1          61 PDSVKGRFTISRDNAKNSLYLQMNSLRAEDTAVYYCTRDLRMSDYFDYWGQGTLVTVSS
HC2          61 PDSVKGRFTISRDSAKNSLYLQMNSLRAEDTAVYYCTRDLRMSDYFDYWGQGTLVTVSS
HC3          61 PDSVKGRFTISRDNSKNTLYLQMNSLRAEDTAVYYCTRDLRMSDYFDYWGQGTMVTVSS
IgVH3-7      61 VDSVKGRFTISRDNAKNSLYLQMNSLRAEDTAVYYCAR
```

B

VL	Full-length (Framework+CDR) Cutoff = 84	Framework Only Cutoff = 88
**85RF45.1**	64	70
**LC1**	85	98
**LC2**	82	96
**LC3**	84	96

VH	Full-length (Framework+CDR) Cutoff = 79	Framework Only Cutoff = 84
**85RF45.1**	75	78
**HC1**	84	92
**HC2**	83	91
**HC3**	84	90

C

Sample ID	KD (M)	kon (1/Ms)	kdis (1/s)	Full X^2	Full R^2
HC1+LC1	5.2E-09	4.5E+05	2.3E-03	0.0192	0.9956
HC1+LC2	5.5E-09	5.0E+05	2.8E-03	0.028	0.9924
HC1+LC3	5.9E-09	4.8E+05	2.9E-03	0.0173	0.9947
HC2+LC1	8.1E-10	4.2E+05	3.4E-04	0.0095	0.9988
HC2+LC2	1.1E-09	3.8E+05	4.3E-04	0.0076	0.9988
HC2+LC3	8.9E-10	4.3E+05	3.8E-04	0.0108	0.9982
HC3+LC1	1.8E-09	4.5E+05	8.1E-04	0.0121	0.9982
HC3+LC2	2.4E-09	4.2E+05	1.0E-03	0.0104	0.9982
HC3+LC3	2.4E-09	4.0E+05	9.7E-04	0.0089	0.9984

Humanization of mAb 85RF45.1. (A) Sequence alignment between mAb 85RF45.1, the closest human germline antibody sequence and the selected light and heavy humanized chains. CDR elements that were kept constant are outline in yellow. Homology to the rat-derived mAb 85RF45.1 sequence is shown in black, and similarity in grey. **(B)** Humanness scores for all humanized chains. **(C)** Affinity determination of humanized 85RF45.1 variants against antigens R0.6C measured by biolayer interferometry.

Supplementary Figure 3

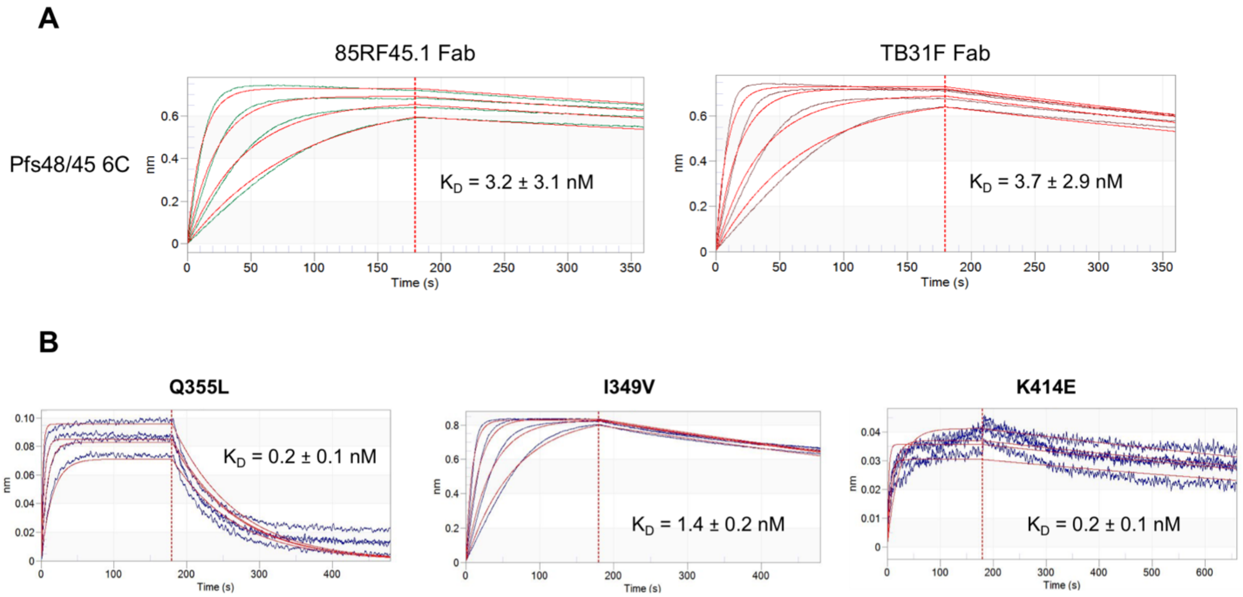

Kinetics of 85RF45.1 and TB31F Fabs binding to Pfs48/45 6C. (**A**) Green and brown lines are representative of raw data, whereas red curves represent global fitting according to a 1:1 model. In both experiments, Pfs48/45 6C is immobilized on Ni-NTA biosensors before being transferred into a solution of Fab for association (left), followed by a dissociation phase in buffer (right). The different sensograms correspond to Fab concentrations of 125 nM, 62.5 nM, 31.3 nM and 15.6 nM. (**B**) Binding affinity of TB31F Fab to Pfs48/45 6C constructs with point mutations representative of sequence polymorphisms in the TB31F epitope. Blue lines are representative of raw data, whereas red curves represent global fitting. The different sensograms correspond to Fab concentrations of 31.3 nM, 15.6 nM, 7.8 nM and 3.9 nM (Q355L and I349V) and of 62.5 nM, 31.3 nM, 15.6 nM and 7.8 nM (K414E). K_D's are indicated with standard deviation and derive from at least two independent measurements.

Supplementary Figure 4

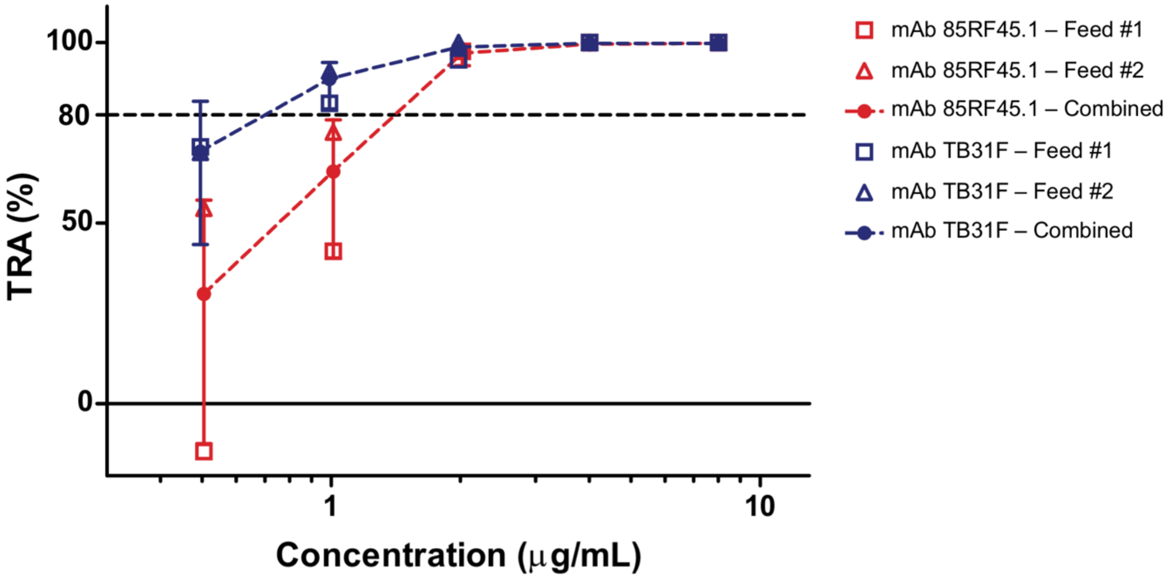

mAbs 85RF45.1 and TB31F have similar transmission reducing activity (TRA) in the SMFA. mAbs were tested at 0.5, 1, 2, 4 and 8 µg mL^{-1} in two separate mosquito feeding experiments (Feed #1 and #2). The best estimate of percentage TRA and 95% confidence intervals are shown in circles and error bars respectively (Combined). The IC_{80} values of mAb 85RF45.1 and mAb TB31F lie between 1-2 µg mL^{-1} and 0.5-1 µg mL^{-1}, respectively.

Supplementary Figure 5

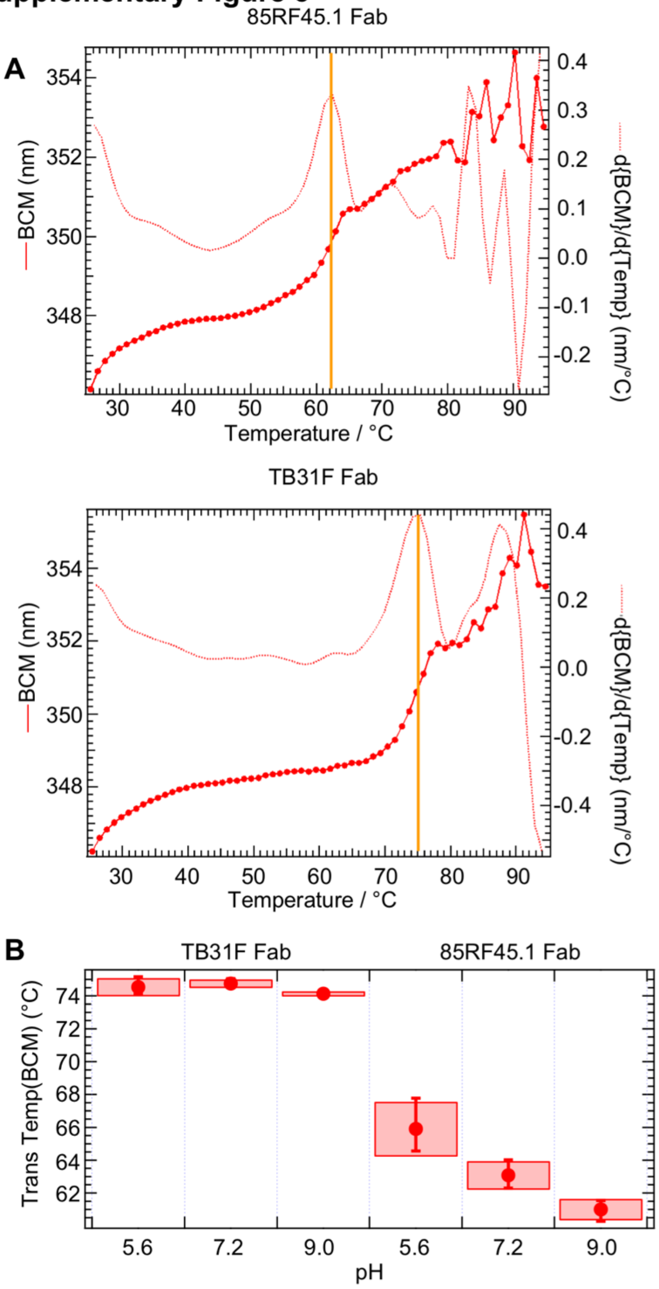

Melting temperature determination for 85RF45.1 and TB31F Fabs. (A) Representative barycentric mean fluorescence (red points and line) measured during a temperature ramp-up for 1 mg mL^{-1} 85RF45.1 (top) and TB31F (bottom) Fabs in 100 mM HEPES pH 7.2, 150 mM NaCl. The melting temperature (yellow line) is determined from the peak of the differential (dotted red line). **(B)** Cumulative T_m determination data for 85RF45.1 (right) and TB31F (left) Fabs measured in triplicates and in buffers of three different pH's. The mean is represented by a dot, the range in the data by lines above and below the mean, and the standard deviation by the shaded box.

Supplementary Figure 6

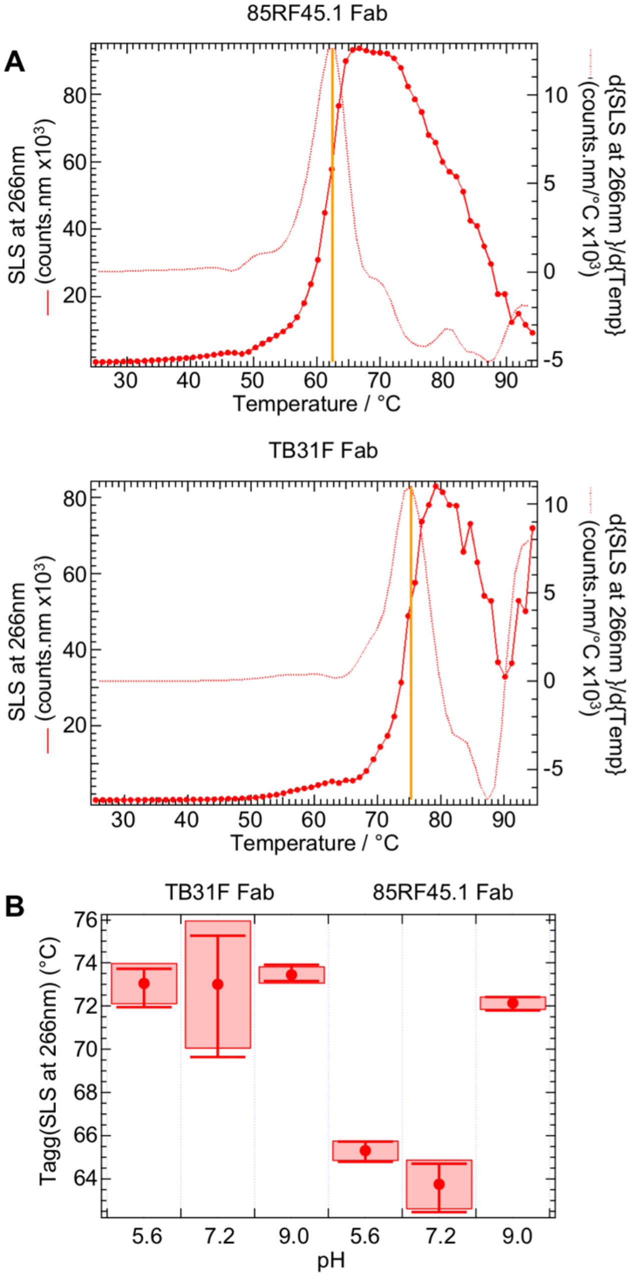

Aggregation temperature determination for 85RF45.1 and TB31F Fabs. (A) Representative static light scattering (SLS) at 266 nm (red points and line) measured during a temperature ramp-up for 1 mg mL^{-1} 85RF45.1 (top) and TB31F (bottom) Fabs in 100 mM HEPES pH 7.2, 150 mM NaCl. The aggregation temperature (yellow line) is determined from the peak of the differential (dotted red line). **(B)** Cumulative T_{agg} determination data for 85RF45.1 (right) and TB31F (left) Fabs measured in triplicates and in buffers of three different pH's. The mean is represented by a dot, the range in the data by lines above and below the mean, and the standard deviation by the shaded box.

Supplementary Figure 7

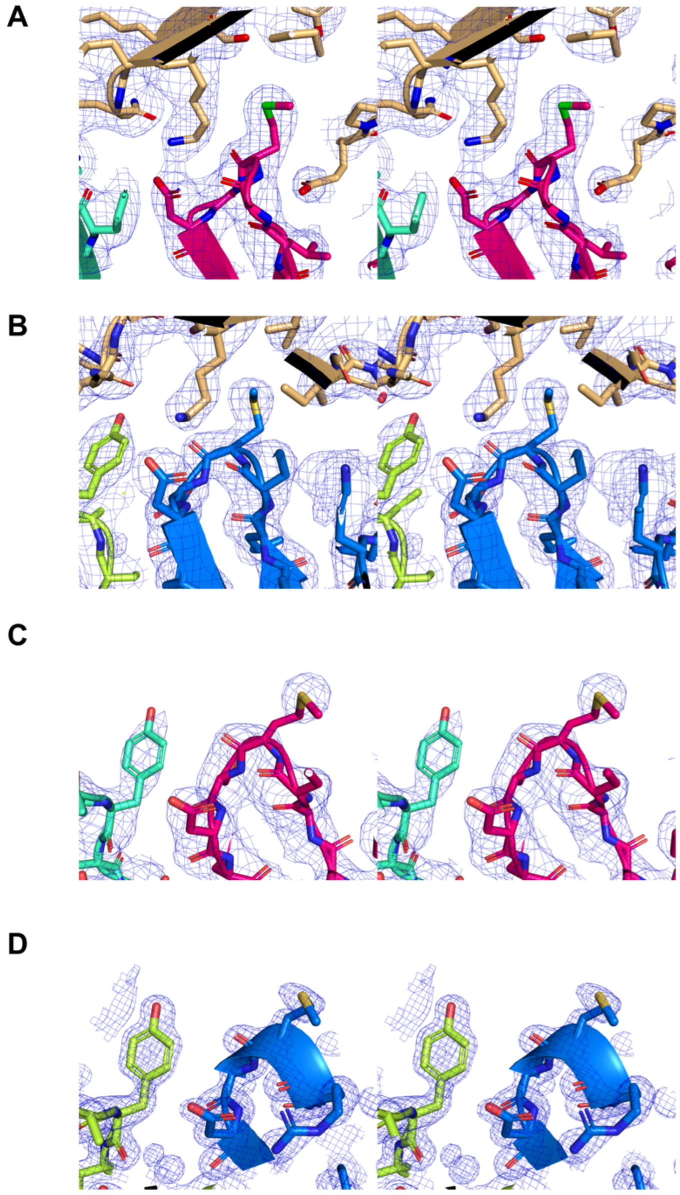

Representative electron density in stereo view for reported crystal structures. Composite omit map contoured at 1σ for **(A)** the antibody-antigen interface of the 85RF45.1 Fab-Pfs48/45 6C co-crystal structure; **(B)** the antibody-antigen interface of the TB31F Fab-Pfs48/45 6C co-crystal structure; **(C)** CDR loop residues of the 85RF45.1 Fab crystal structure; and **(D)** CDR loop residues of the TB31F Fab crystal structure.

Supplementary Table 1: Data collection and refinement statistics.

	Pfs48/45 6C – 85RF45.1 Fab	Pfs48/45 6C – TB31F Fab	85RF45.1 Fab	TB31F Fab
**Data Collection**				
Wavelength (Å)	0.979490	0.979490	0.979490	0.999977
Space group	$P2_1\ 2_1\ 2_1$	$P2\ 2_1\ 2_1$	$P2_1\ 2_1\ 2_1$	$P2_1\ 2_1\ 2_1$
Cell dimensions				
a, b ,c (Å)	54.51, 74.45, 321.89	51.99, 120.91, 177.56	71.88, 86.52, 139.87	55.68, 70.72, 115.36
α, β, γ (°)	90, 90, 90	90, 90, 90	90, 90, 90	90, 90, 90
Resolution (Å)[a]	48.7 – 2.7 (2.8 – 2.7)	49.9 – 2.6 (2.7 – 2.6)	43.4 – 3.15 (3.25 – 3.15)	44.7 – 1.5 (1.6 – 1.5)
No. molecules in ASU	2	2	2	1
No. total observations	489,243	467,201	202,599	956,274
No. unique observations	37,228 (3,756)	35,359 (3,678)	15,740 (1,394)	73,693 (12,770)
Multiplicity[a]	13.1 (13.4)	13.2 (12.9)	12.9 (11.9)	13.0 (13.1)
R_{merge} (%)[a]	15.4 (80.6)	25.8 (82.4)	29.2 (83.0)	9.4 (80.9)
R_{pim} (%)[a]	4.5 (22.7)	7.3 (23.8)	8.4 (24.6)	2.7 (23.0)
$\langle I/\sigma I \rangle$	14.4 (1.7)	8.9 (1.6)	7.2 (1.5)	15.9 (1.8)
$CC_{1/2}$	99.7 (62.6)	98.5 (65.5)	99.3 (73.0)	99.9 (62.8)
Completeness (%)	100.0 (100.0)	99.9 (100.0)	99.8 (99.9)	100.0 (100.0)
Wilson B-value (Å^2)	64.2	33.7	56.3	20.2
**Refinement**				
Non-hydrogen atoms	8,799	8,636	6,542	3,807
Macromolecule	8,708	8,459	6,542	3,282
Solvent	91	177	-	525
R_{work}/R_{free} (%)	23.2/27.3	20.2/24.0	23.6/27.8	17.2/18.7
Rms deviations from ideality				
Bond lengths (Å)	0.003	0.005	0.004	0.006
Bond angle (°)	0.615	0.741	0.783	0.897
Ramachandran plot				
Favoured regions (%)	94.3	96.3	97.4	98.8
Allowed regions (%)	5.3	3.2	2.6	1.2
B-factors (Å^2)				
Average B-factors	111.6	38.4	69.5	26.8
Average macromolecule	112.2	38.5	69.5	25.1
Average solvent	87.7	40.4	-	37.7

[a]Values in parentheses refer to the highest resolution bin.

Supplementary Table 2: 85RF45.1-Pfs48/45 and TB31F-Pfs48/45 interactions.

Pfs48/45 Residue (BSA Å^2 85RF45.1/TB31F)	Interaction Type	85RF45.1 Residue	Interaction Type	TB31F Residue
**Asp321 (10.6/16.9)**				
Asp	VDW	L-Gly29, L-Ser30	VDW	L-Gly29, L-Ser30
**Ser322 (0/30.7)**				
Ser			VDW	L-Tyr32, L-Arg50
Ser^{OG}			HB	L-Tyr32^{OH}
**Pro345 (0.7/5.0)**				
Pro	VDW	L-Thr93	VDW	L-Thr93
**Gly346 (12.6/10.8)**				
Gly	VDW	L-Thr93, H-Tyr58	VDW	L-Thr93, H-Tyr58
Gly^{O}	HB	H-Tyr58^{OH}		
**Asp347 (83.1/78.0)**				
Asp	VDW	L-Tyr91, L-Thr93, H-Trp33, H-Thr56, H-Tyr58, H-Arg97	VDW	L-Tyr91, L-Thr93, H-Trp33, H-Thr56, H-Tyr58, H-Arg97
Asp^{OD1}	HB	H-Tyr58^{OH}	HB	H-Tyr58^{OH}
Asp^{OD2}	HB	H-Arg97^{NH1}, L-Tyr91^{OH}		
Asp^{OD2}	SB	H-Arg97^{NH1}	SB	L-Tyr91^{OH}, H-Arg97^{NE}, H-Arg97^{NH1}
**Ile348 (17.5/17.6)**				
Ile	VDW	H-Trp33	VDW	H-Trp33
**Ile349 (35.6/37.2)**				
Ile	VDW	H-Trp33, H-Arg97, H-Met98	VDW	H-Trp33, H-Arg97, H-Met98
**Pro350 (5.2/4.3)**				
Pro	VDW	H-Asn52A	VDW	H-Asn52A
**Asp351 (95.1/94.2)**				
Asp	VDW	H-Ser52, H-Asn52A, H-Ile53, H-Gly54, H-Gly55, H-Thr56	VDW	H-Trp33, H-Ser52, H-Asn52A, H-Ile53, H-Gly54, H-Gly55, H-Thr56
Asp^{OD2}	HB	H-Ser52^{OG}, H-Asn52A^{N}, H-Ile53^{N}, H-Gly55^{N}	HB	H-Ser52^{OG}, H-Asn52A^{N}, H-Ile53^{N}, H-Gly55^{N}

**Phe354 (11.3/17.1)**				
Phe	VDW	H-Ile53	VDW	H-Ile53
**Gln355 (46.1/43.1)**				
Gln	VDW	H-Asn30, H-Asn31, H-Asn52A, H-Ile53	VDW	H-Asn30, H-Asn31, H-Asn52A, H-Ile53
Gln^{OE1}	HB	$H\text{-}Asn52A^{ND2}$	HB	$H\text{-}Asn52A^{ND2}$
Gln^{NE2}	HB	$H\text{-}Asn30^{O}$	HB	$H\text{-}Asn30^{O}$
**Tyr357 (0/5.8)**				
Tyr			VDW	H-Met98
**Leu364 (90.7/0)**				
Leu	VDW	L-Arg50, H-Met98, H-Ser99		
**Glu365 (87.3/21.3)**				
Glu	VDW	L-Arg50, H-Leu96, H-Arg97, H-Met98, H-Ser99	VDW	L-Arg50, H-Met98
Glu^{OE1}	HB	$H\text{-}Ser99^{OG}$		
Glu^{OE2}	HB	$H\text{-}Arg97^{N}$, $H\text{-}Met98^{N}$, $H\text{-}Ser99^{N}$		
**Pro366 (6.0/0)**				
Pro	VDW	H-Met98		
**Ser367 (30.5/0)**				
Ser	VDW	H-Asn31, H-Tyr32		
**Ile369 (39.6/50.2)**				
Ile	VDW	H-Asn31, H-Asn52A	VDW	H-Asn31, H-Asn52A
**Tyr371 (31.9/36.2)**				
Tyr	VDW	H-Asn30, H-Asn31, H-Ile53	VDW	H-Asn30, H-Ile53
Tyr^{OH}			HB	$H\text{-}Asn30^{ND2}$
**Glu385 (0/1.5)**				
Glu			VDW	H-Gly55
**Asp390 (25.5/17.7)**				
Asp	VDW	L-Thr93	VDW	L-Thr93
**Lys392 (42.0/48.7)**				
Lys	VDW	H-Thr56, H-Tyr58,	VDW	L-Thr93, H-Thr56, H-Tyr58
**Lys394 (33.0/29.3)**				
Lys	VDW	H-Gly55	VDW	H-Gly55
Lys^{NZ}	HB	$H\text{-}Gly55^{O}$	HB	$H\text{-}Gly55^{O}$
**Ile411 (10.7/12.1)**				
Ile	VDW	H-Met98	VDW	H-Met98

**Lys413 (90.8/87.1)**				
Lys	VDW	L-Tyr32, L-Tyr91, L-Thr93, H-Arg97, H-Met98, H-Ser99, H-Asp100	VDW	L-Tyr32, L-Tyr91, H-Arg97, H-Met98, H-Ser99, H-Asp100
Lys^{NZ}	HB	$H\text{-}Asp100^{OD2}$	HB	$H\text{-}Asp100^{OD2}$
Lys^{NZ}	SB	$H\text{-}Asp100^{OD1}$, $H\text{-}Asp100^{OD2}$	SB	$H\text{-}Asp100^{OD1}$, $H\text{-}Asp100^{OD2}$
**Lys414 (20.2/12.2)**				
Lys	VDW	L-Thr93	VDW	L-Thr93
Lys^{NZ}	HB	$L\text{-}Thr93^{OG1}$		
**Asp415 (79.2/59.8)**				
Asp	VDW	L-Ser30, L-Asn31, L-Tyr32, L-Tyr91, L-Ser92, L-Thr93, H-Asp100	VDW	L-Ser30, L-Asn31, L-Tyr32, L-Ser92, L-Thr93, H-Asp100
Asp^{OD1}	HB	$L\text{-}Asn31^{ND2}$	HB	$L\text{-}Asn31^{ND2}$
**Lys416 (131.1/124.5)**				
Lys	VDW	L-Gly29, L-Ser30, L-Asn31, L-Tyr32, L-Asp51	VDW	L-Ile28, L-Gly29, L-Ser30, L-Asn31, L-Tyr32, L-Asp51, L-Arg66B
Lys^{O}			HB	$L\text{-}Tyr32^{OH}$
Lys^{N}	HB	$L\text{-}Ser30^{O}$	HB	$L\text{-}Ser30^{O}$
Lys^{NZ}	HB	$L\text{-}Asp51^{OD2}$	HB	$L\text{-}Asp51^{OD2}$
Lys^{NZ}	SB	$L\text{-}Asp51^{OD2}$	SB	$L\text{-}Asp51^{OD2}$, $L\text{-}Asp51^{OD2}$
**Ser418 (7.0/5.4)**				
Ser	VDW	H-Met98	VDW	H-Met98

Supplementary Table 3: SMFA luciferase data. Values are relative light units (RLU) after background subtraction. Background is the mean luciferase signal of 8 wells to which only substrate was added. Each value represents the readout of a pool of 5 mosquitoes.

		Feed #1				Feed #2			
		pool 1	pool 2	pool 3	pool 4	pool 1	pool 2	pool 3	pool 4
Control	feeder 1	4.5	8.5	12.5	9.5	61.4	105.4	209.4	100.4
	feeder 2	5.5	9.5	18.5	14.5	102.4	66.4	267.4	43.4
mAb 85RF45.1	0.5 µg/ml	23.5	5.5	6.5	11.5	63.4	67.4	51.4	37.4
	1.0 µg/ml	10.5	3.5	4.5	5.5	32.4	26.4	7.4	52.4
	2.0 µg/ml	0	0.5	1.5	0	5.4	0	0	7.4
	4.0 µg/ml	0	0.5	0	0.5	0	0	1.4	0
	8.0 µg/ml	0	0	0	0.5	0	0	0	0.4
mAb TB31F	0.5 µg/ml	2.5	0.5	3.5	5.5	26.4	75.4	0	46.4
	1.0 µg/ml	2.5	1.5	2.5	0.5	1.4	16.4	15.4	4.4
	2.0 µg/ml	0	1.5	1.5	0	0	2.4	0	0
	4.0 µg/ml	0	0	0	0	0	0	0.4	0.4
	8.0 µg/ml	0	0.5	0	0	0	0	0	0.4

Supplementary References

1. Tonkin ML*, et al.* Structural and biochemical characterization of Plasmodium falciparum 12 (Pf12) reveals a unique interdomain organization and the potential for an antiparallel arrangement with Pf41. *J Biol Chem* **288**, 12805-12817 (2013).

2. Parker ML, Peng F, Boulanger MJ. The Structure of Plasmodium falciparum Blood-Stage 6-Cys Protein Pf41 Reveals an Unexpected Intra-Domain Insertion Required for Pf12 Coordination. *PLoS One* **10**, e0139407 (2015).

3. Crawford J, Lamb E, Wasmuth J, Grujic O, Grigg ME, Boulanger MJ. Structural and functional characterization of SporoSAG: a SAG2-related surface antigen from Toxoplasma gondii. *J Biol Chem* **285**, 12063-12070 (2010).
